# Quantifying the stabilizing effects of protein–ligand interactions in the gas phase

**DOI:** 10.1038/ncomms9551

**Published:** 2015-10-06

**Authors:** Timothy M. Allison, Eamonn Reading, Idlir Liko, Andrew J. Baldwin, Arthur Laganowsky, Carol V. Robinson

**Affiliations:** 1 Department of Chemistry, University of Oxford, South Parks Road, Oxford OX1 5QY, UK

## Abstract

The effects of protein–ligand interactions on protein stability are typically monitored by a number of established solution-phase assays. Few translate readily to membrane proteins. We have developed an ion-mobility mass spectrometry approach, which discerns ligand binding to both soluble and membrane proteins directly via both changes in mass and ion mobility, and assesses the effects of these interactions on protein stability through measuring resistance to unfolding. Protein unfolding is induced through collisional activation, which causes changes in protein structure and consequently gas-phase mobility. This enables detailed characterization of the ligand-binding effects on the protein with unprecedented sensitivity. Here we describe the method and software required to extract from ion mobility data the parameters that enable a quantitative analysis of individual binding events. This methodology holds great promise for investigating biologically significant interactions between membrane proteins and both drugs and lipids that are recalcitrant to characterization by other means.

## Abstract (web summary)

Relatively few techniques can quantitatively measure the effect of ligands on membrane protein stability. Here the authors demonstrate the use of ion-mobility mass spectrometry to accurately measure and quantify ligand-induced protein stabilization in the gas phase.

## Graphical abstract

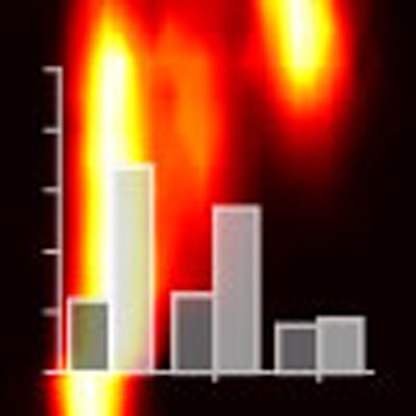

To understand the function of biomolecules, it is crucial to be able to both identify their binding partners and characterize the strength of the interactions. This has led to the employment of a very diverse range of biophysical techniques to study ligand binding, which are generally based on spectroscopic properties of the molecules in question or the heat change associated with the binding process. These measurements are typically ensemble measurements where the observable contains contributions from both the free and bound states.

Mass spectrometry (MS) has proven to be a useful approach for the assessment of protein oligomeric state, binding stoichiometry and the structure and stability of intact protein–ligand complexes^1^. The coupling of ion mobility (IM) is further enhancing the capabilities of MS by providing novel insight into protein structure and dynamics, and for drug discovery and development^2^,^3^. Here we describe a method involving the characterization of the gas-phase stability of proteins using IM-MS. The method quantifies the resistance of proteins to unfolding in the gas phase, which can be significantly modulated by ligand binding. By measuring the change in this stability, specific ligand interactions can be identified even in systems as complex as membrane proteins solubilized in various detergent and lipid assemblies. Individual binding states can be uniquely identified and the effects of ligand binding measured with unprecedented sensitivity. We show that in the case of membrane proteins, specific lipid binding can be readily distinguished from the background signal of detergents that otherwise complicate analysis, demonstrating the significant potential of this method.

In our approach, weakly bound molecules such as detergent or other solubilizing molecules are first removed from the protein in the gas phase, before the stability analysis. Binding of a ligand is evidenced by a change in the mass of the protein complex, which is readily determined under non-denaturing MS conditions. The effects on the protein stability due to ligand binding are then investigated using collision-induced unfolding inside the mass spectrometer. In this process, protein ions are accelerated through a collision cell in the presence of a neutral gas and undergo collisional activation^4^. The activation causes the protein to change conformation, typically by partial unfolding, yet nevertheless can be sufficiently gentle to retain quaternary structure^4^,^5^. The extent of activation can be directly influenced by changing the voltage used to accelerate the ions into the collision cell. Importantly, by having the collisional activation occur before entry into the mobility cell of the mass spectrometer, the averaged gas-phase collision cross-section (CCS) values (effectively the size) of both folded and unfolded ions can be obtained at a single *m/z* value, enabling conformational changes to be detected and quantified. By following the unfolding as a function of the accelerating voltage, the gas-phase stability of the complex can be determined, in a manner that is analogous to how protein stability is inferred from its behaviour in denaturant assays. Ligand binding manifests itself as a change in protein–ligand stability relative to the ligand-free form, a property exploited in our method.

Previous studies have observed the stabilizing effects of ligand binding in the gas phase by collision-induced unfolding^3^,^6^,^7^, with a variety of approximate methods employed to assess differences in stability. These include an approximation of the midpoint between the smallest- and largest-sized species observed, or noting qualitatively different patterns in the unfolding trajectory. These analyses have provided the inspiration for the method implemented in the software presented here, which is based on the work of Hyung *et al*.^7^, where the stability of folded native-like ions, in different bound states, were tracked as a function of collisional activation. However, exploiting the rich information in collision-induced unfolding measurements has been hampered by the challenges associated with dealing with the high volume of experimental data and the lack of suitable quantitative means to extract robust and reliable binding parameters. To address these concerns, here we present experimental methodology coupled with novel software for processing and rigorously analysing high volumes of IM-MS data. This software fits the collision-induced unfolding data to an unfolding model that provides an accurate description of the effects of ligand binding on gas-phase protein stability and is proving uniquely adept at analysing challenging protein complexes such as those encountered in biological membranes^8^.

## Results

### Optimization of MS ligand-binding conditions

**Figure 1:**
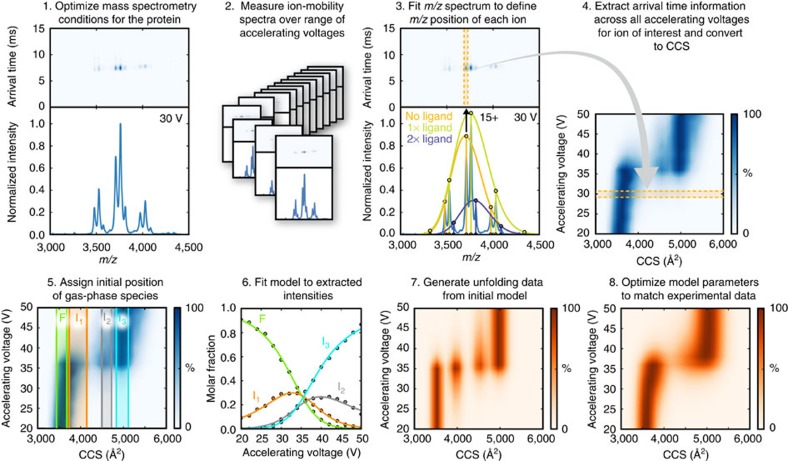
Schematic of the gas-phase protein unfolding experiment and modelling process. Data shown are for human transthyretin with up to two molecules of L-thyroxine bound, with the unfolding plot generated and modelled for the unbound 15+ ion.

An overview of our method is presented in Fig. 1. The first step is an empirical optimization of the non-denaturing MS conditions for each protein to be studied (Fig. 1, step 1)^9^. It is crucial to balance the removal of complicating nonspecific adducts and maintaining a folded conformation. In the case of membrane proteins, this normally involves screening different solubilizing detergents^10^ to establish optimal resolution between the unbound and ligand-bound forms in the mass spectrum. In addition, for membrane proteins it is necessary, in general, to operate at higher accelerating voltages to first remove the detergent micelle^11^. Fortuitously, the protein is effectively protected from premature unfolding by the presence of the protective coating afforded by the micelle^12^. The folded state of the protein in the gas phase is assessed by comparison with the theoretical CCS, calculated, for example, from the atomic coordinates of an X-ray crystallography or other model^13^,^14^,^15^. It is important that the measured CCS is consistent with the theoretical CCS, as otherwise the protein may already be either unfolded or collapsed and hence unsuitable for study. We note that different membrane proteins require different solubilizing detergents to satisfy this requirement^10^,^11^. When optimized conditions are established, ligands are introduced and the protein–ligand concentration ratio adjusted to preserve mass spectral quality at the low activation energies that preserve the folded conformation. Data can then be acquired and analysed as described below.

### Recording and visualization of IM mass spectra

Collision-induced unfolding experimental data consist of a set of two-dimensional IM mass spectra recorded for a range of increasing accelerating voltages (Fig. 1, step 2). Each set of accelerating voltage measurements are carried out on the same solution using a minimum number of nanoflow needles to reduce variability in electrospray conditions. The process should be repeated a minimum of three times, to average out random variability from the electrospray process, so that robust parameters can be extracted. The arrival time distribution for ions of a particular *m/z* value at a range of accelerating voltages are extracted (Fig. 1, step 3) and then stacked to produce a gas-phase unfolding plot (Fig. 1, step 4). The plots show how collisional activation, controlled by the application of accelerating voltage, changes the size of an ion. These data are then quantitatively analysed by our software.

### IM data analysis to quantify gas-phase stability

Our method analyses the change in size of an ion as a function of accelerating voltage according to a model of equilibrium unfolding, a method analogous to that used to analyse protein stability in denaturant assays in solution. Any changes in the observed stability in the presence of ligand can be directly attributed to the binding of the ligand. We accomplish this in a semi-automatic manner using our software package *PULSAR* (Protein Unfolding for Ligand Stability and Ranking) (Supplementary Movie 1). This software can directly import data generated from both travelling-wave and drift-tube IM cell instruments without format conversion, create and apply CCS calibrations, construct gas-phase unfolding plots and apply an equilibrium unfolding model to quantify ligand-induced protein stabilization.

To assemble an unfolding plot, the *m/z* value for each ion of interest needs to be determined so that the corresponding window of the arrival-time distribution can be extracted (Fig. 1, step 3). To do this, the software performs *m*/*z* peak fitting, where a set of Gaussian functions is used to model the mass spectra. Briefly, the overall charge-state distribution of a species is represented by one Gaussian function and each peak within this envelope is modelled by another Gaussian function^16^. This fit provides the centroid values for every *m/z* peak in the mass spectra, which allows the arrival-time distribution for individual ions to be extracted at each measured accelerating voltage, and then assembled into an unfolding plot (Fig. 1, step 4).

During a collision-induced unfolding experiment, as the accelerating voltage is increased, ions may transition through a series of intermediates in which the size of the ion (measured as the arrival time) can decrease and/or increase, relative to that of the initial folded state, due to and/or unfolding of the protein. Depending on the system under study as well, the number of species that can be resolved depends on the IM resolution. At particular accelerating voltages, a species, be it folded or an intermediate, will begin to disappear simultaneous with another species appearing, such that at any given accelerating voltage, the fraction of the protein in each state is measured. By fitting these data to an unfolding model, the stability of each state can be determined. Our experience indicates that stabilizing ligands increase the accelerating voltages at which transitions in an unfolding plot take place and thus increase the resistance of proteins to unfolding.

### Description and application of unfolding model

To quantify gas-phase stability we use an equilibrium unfolding model, similar to those used for solution studies^17^,^18^. In the simplest case of a two-state system, this model assumes equilibrium between two species, F (folded) and U (most-unfolded form), described by the equilibrium constant *K*_eq_:




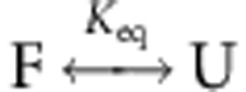




where the stability of a species is given by the equation:




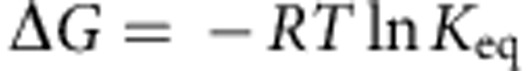




From our measurements of the relative intensities of each species, we can calculate the fraction of each species present at each accelerating voltage. Analogously to denaturant studies in solution, we assume that the accelerating voltage (*V*) is related linearly to destabilization such that the free energy can be expressed as:




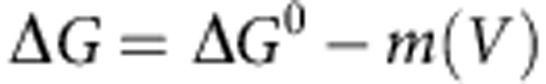




From this model, we obtain the effective stability of each species at zero accelerating voltage (Δ*G*^0^) and the proportionality coefficient, *m*. The accelerating voltage at the midpoint of the unfolding transition of a species will then be at Δ*G*^0^/*m*. As we observe a number of species, we model the unfolding as a linear pathway by invoking successively large numbers of intermediate unfolded forms.

Our justification of the equilibrium unfolding model is twofold. By holding the ions in the mobility cell for different lengths of time, we can assess whether or not the species move further down their unfolding pathway as a function of time. If the unfolding process were irreversible in a manner that depends purely on the time spent at a given activation level, then the ion would progress steadily and irreversibly to the unfolded state the longer it is held in the mobility cell. By contrast, we observe that the distribution of unfolded states does not depend appreciably on time^8^, demonstrating that this type of irreversible unfolding is not occurring in the instrument. Second, the data we obtain for unfolding are sigmoidal in character, which is the expected form for equilibrium-type models, and are not expected for models describing irreversible unfolding. Taken together, the unfolding process with collisional activation appears to behave in a manner that appears to be reversible to some extent. Ultimately, the method relies on observing changes in the unfolding plots and so our conclusions do not rely on the underlying mechanism of unfolding being reversible.

Applying this model to the unfolding data is a three-stage process: (1) assigning the initial positions of each species in the unfolding plot, (2) generating initial model parameters, to be used for (3) fitting of the model to the experimental data. First, species (the folded state, an intermediate and/or the most unfolded form) are assigned in the unfolding plot: each species is given an initial position and width (Fig. 1, step 5). Second, to generate initial Δ*G*^0^- and *m*-values, the intensity of each species within these boundaries is summed at each accelerating voltage to give an estimate of the fractional amount of each species, to which the model is initially fitted (Fig. 1, step 6). Finally, we construct a theoretical unfolding plot using the unfolding model, seeded with the Δ*G*^0^- and *m*-values from the simpler fit (Fig. 1, step 7), which is fitted to the experimental data (Fig. 1, step 8). The software lets the user assign the initial position and widths of the species, and then automatically extracts and fits the data for the rest of the process. The data should be simulated with increasing numbers of intermediates. The addition of an extra intermediate increases the number of parameters in the model. An F-test should then be performed after each addition, to determine whether the inclusion of each additional intermediate is statistically justified. This results in the equilibrium unfolding model accurately reproducing the experimental unfolding plot, from which gas-phase ligand-induced protein stabilization can be calculated.

### Calculation of gas-phase stability metric

Having used the equilibrium unfolding model to generate a theoretical unfolding plot, which is fitted to the experimental data, it remains to use this model to describe ligand-induced protein stabilization. In applications of equilibrium unfolding models it is more conventional to use the free energy of unfolding and the proportionality constant (*m*), to describe the conformational stability of different species. Here we find that the accelerating voltage corresponding to the midpoint value of a species, calculated as Δ*G*^0^/*m*, is a better metric when measured over multiple repeated runs. Random processes in the electrospray process appear to have a disproportionate effect on Δ*G*^0^ and *m*, but largely cancel out when the quantity Δ*G*^0^/*m* is interrogated.

To calculate the ligand-induced protein stabilization, we consider all transitions of a ligand-bound protein between folded and unfolded species, and compare the midpoint of each of these species with the equivalent midpoint of the ligand-free form. We then sum these differences to generate the stabilization. Although it does not affect the comparison of different ligands, we normalize the stabilization value by the number of transitions. That is, ligand-induced protein stabilization is calculated as the average change in accelerating voltage per transition. In addition, this value can also be converted into a ‘laboratory energy’ by multiplying it by the charge state of the ion to account for the charge-dependent experience of an ion to the accelerating voltage^19^. Although the stabilization in these units provides a relative sense of stabilization, it may be possible to gain further insight into the physical processes associated with unfolding by conversion to centre-of-mass collision energies. For the purposes of assessing stability, we prefer to avoid the assumptions inherent in this conversion. Notably, errors in the stabilization values calculated by bootstrapping analysis closely match those from replica measurements (Table 1), indicating the absence of systematic errors and the reproducibility of the measurements. We recommend that the bootstrapping analysis (provided with the software) is performed routinely in addition to replica measurements, to give confidence to the interpretation of results. The resulting values of the stabilization metric can then be compared for different ligands, or different numbers of the same bound ligand, to rank their protein-stabilizing effects.

The electrospray process generates ions of the same mass but with different charge states, of varying intensity, which gives rise to charge state distributions in the mass spectrum. This means there is a choice of charge state to use for the analysis of gas-phase stability. The charge state chosen should be that with the highest intensity in the IM mass spectra that, at the lowest accelerating voltages used, remains in a folded conformation. Using low-intensity data results in models with poorer fits and subsequently larger errors in the calculated protein stabilization metrics.

### Effects of ligand binding on soluble protein stability

**Figure 2:**
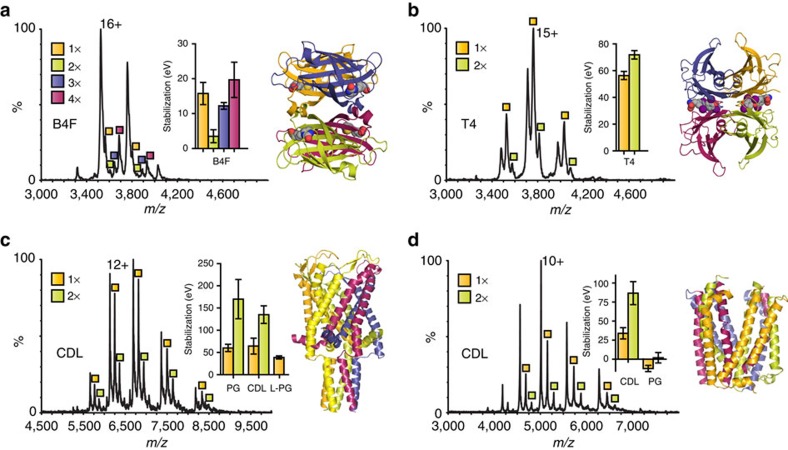
Gas-phase stabilization of soluble and membrane proteins by ligand molecules. For each system, a typical mass spectrum is shown, with the derived stabilization of the protein by the ligand(s) and a representative crystal structure of the protein. (**a**) Streptavidin with B4F (stabilization for 16+ charge state, PDB entry 1STP with bound biotin shown as spheres); (**b**) transthyretin with L-thyroxine (T4) (stabilization for 15+ charge state, PDB entry 2ROX with bound T4 shown as spheres); (**c**) *S. aureus* MscL with the lipids L-α-PG, CDL and lysyl-PG (stabilization for 12+ charge state) (structure shown of *M. tuberculosis* MscL, PDB entry 2OAR). (**d**) The multidrug and toxic compound extrusion protein from *P. furiosus* with CDL and PG (stabilization for 11+ charge state, PDB entry 3VVN). Reported are average and s.e.m. from repeated measurements (*n*=*3*).

To explore the utility of our approach we first analysed the protein-stabilizing effects of ligand binding to well-characterized soluble protein–ligand systems. The first of these was streptavidin and biotin-4-fluorescein (B4F)^20^. The mass spectrum of streptavidin with B4F clearly shows unbound protein and the binding of up to four B4F molecules, consistent with the number of biotin-binding sites on the protein (Fig. 2a). Having resolved these ligand-bound states, we established through comparison of the experimentally determined CCS values with that calculated from the crystal structure that streptavidin is folded at the lowest accelerating voltages. We then collected IM mass spectra over a range of accelerating voltages and followed the remaining steps (three to eight) of the protocol using our software. We observe that the binding of B4F to streptavidin increases its gas-phase stability. This is entirely consistent with previous studies, which have observed increased tetramer stability on biotin binding^21^. Interestingly, the change in stability of the different ligand-bound forms varies nonlinearly, suggesting we can resolve subtleties in the multi-ligand binding that are otherwise exceedingly challenging to infer^22^.

To further validate the methodology, we took a second soluble protein, transthyretin, which is known to bind two molecules of L-thyroxine. We observed precisely this in the mass spectrum (Fig. 2b). As before, we established that the CCS values at low accelerating voltages match that calculated for the crystal structure. Comparison of the unfolding trajectories and computation of the average ligand-induced protein stabilization shows that binding of one and two thyroxine molecules stabilizes transthyretin. Binding of the second thyroxine molecule elicits a smaller increase in stability than binding of the first, which is entirely consistent with the established negative cooperativity of binding, determined using equilibrium dialysis and fluorescence quenching^23^,^24^,^25^.

The extent (and resolution) of stabilization in the gas phase is protein dependent, however, predominantly due to differences in ion size, charge and energy, ligand type and number, and structural features of the protein that resist unfolding, making it difficult to predict *a priori*. Similarly, predicting stabilities and dissociation constants of protein–ligand complexes in solution remains a challenging prospect. The strength of this method is that for an individual protein, the relative extents of ligand-induced stabilization in the gas phase can be used to compare different ligands or different ligand-bound states present in solution.

### Stability of membrane proteins is influenced by bound lipid

Having established that the approach reports on ligand-induced changes in protein stability for soluble proteins, we sought to test the method in the more formidable case of membrane proteins. Although many methods exist for measuring the effect of ligand binding on the stability of soluble proteins, application of these to membrane proteins is challenging. Our method, however, is able to robustly characterize the effect of ligand binding on the stability of membrane proteins. Extending our previous preliminary work^8^, we selected three further membrane proteins for study: two α-helical proteins, the mechanosensitive channel of large conductance from *Staphylococcus aureus* (*Sa*MscL) and the multi-antimicrobial extrusion protein from *Pyrococcus furiosus* (*Pf*MATE), and the β-barrel outer-membrane protein OmpF from *Escherichia coli* (*Ec*OmpF).

We previously identified that the mechanosensitive channel of large conductance from *Mycobacterium tuberculosis* (*Mt*MscL) is stabilized by different types of lipids to a similar extent, but also that phosphatidylinositol, which is crucial for mechanosensitivity of the channel^26^, avidly bound to the protein under the experimental conditions used^8^. The *S. aureus* membrane is primarily composed of cardiolipin (CDL), phosphatidylglycerol (PG) and the cationic phospholipid lysyl-PG^27^; thus, we were interested to assess whether and to what extent *Sa*MscL is stabilized by these lipids. Following the protocol defined earlier, conditions were optimized for MS and the CCS values of the protein at low accelerating voltages were found to be similar to that of the crystal structure of the homologous *Mt*MscL. The binding of up to two molecules of PG was clearly discerned and the increase in protein stabilization with lipid binding was found to be nonlinear and potentially cooperative (Fig. 2c). Only one bound molecule of lysyl-PG was observed, with an average stabilizing effect similar to that of one PG molecule. Up to two CDL molecules were observed to bind to the *Sa*MscL, with an additive effect on protein stability, implying the binding events are independent and not cooperative. Although the binding of a single lipid to *Sa*MscL has an approximately equal stabilizing effect independent of the type of lipid, the discrimination of bound states reveals differences in the cooperativity of lipid-induced protein stabilization. This is in contrast to *Mt*MscL, for which the binding of multiple lipids showed no cooperative protein stabilization effects^8^, indicating that MscL proteins from different species may respond to lipid binding in different ways.

The second membrane protein we investigated was the *Pf*MATE. *P. furiosus* is an archaea, the native membrane lipids of which differ to those in the expression host that we used, *E. coli*, in three principle ways: archaeal lipids have phytanyl tails and ether linkages between the head group and the tail, whereas bacterial lipids have fatty acid tails and ester linkages, and in addition the stereochemistry of the head groups are also different^28^. Nevertheless, under non-optimized purification conditions, *Pf*MATE will co-purify bound to CDL from the *E. coli* membrane. Using lipid-free purified *Pf*MATE, similar to the experiments performed for *Sa*MscL above, when *E. coli* CDL is added to the protein solution, we observe binding of two CDL molecules under optimized MS conditions. The binding of CDL to *Pf*MATE has an additive effect on stability (Fig. 2d). Interestingly, performing the same experiment with PG, although we observe binding to *Pf*MATE, there is a slight destabilization for one lipid bound and net no change for two lipids bound (Fig. 2d). This indicates specific lipids have different stabilizing effects on this protein.

**Figure 3:**
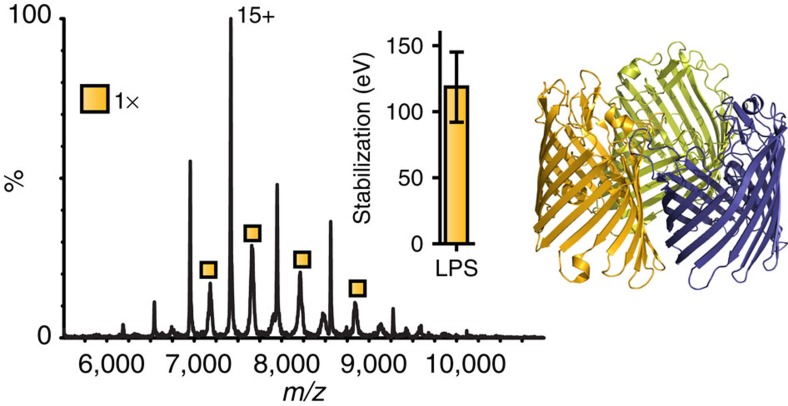
Mass spectrum of the outer membrane protein OmpF from *E. coli* and gas-phase stabilization by rough lipopolysaccharide (LPS; for 16+ charge state). The protein structure is of *E. coli* OmpF (PDB entry 2ZFG). Reported are average and s.e.m. from repeated measurements (*n*=*3*).

The third membrane protein studied was *Ec*OmpF, which is a trimeric β-barrel protein. This protein was purified bound to rough lipopolysaccharide, a constituent of the outer membrane of the *E. coli* in which this protein was expressed^29^. We observe stabilization of the protein complex by rough lipopolysaccharide (Fig. 3), implying that this outer-membrane lipid is bound *in vivo*. The results with these three membrane proteins demonstrate the power of the method to discern the stabilizing effects of binding different types of lipids.

### Comparison of gas-phase with solution-based assays

**Figure 4:**
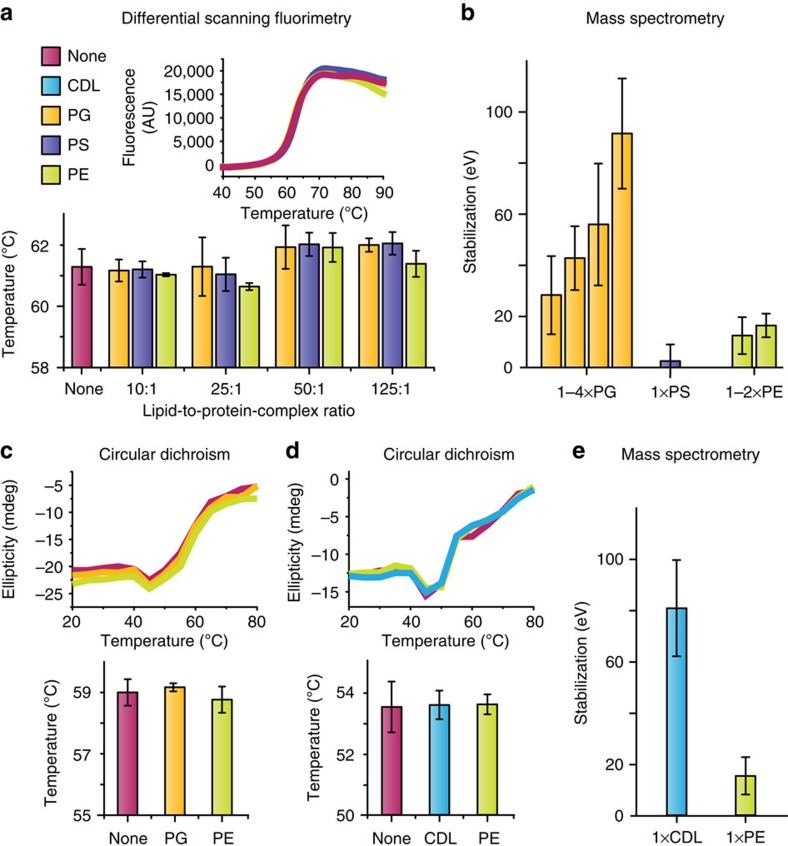
Comparison of solution- and gas-phase unfolding experiments with two membrane proteins, ammonia channel (AmtB) and aquaporin Z (AqpZ), in the presence of different lipids. (**a**) Thermal denaturation (*T*_m_) measurements obtained by DSF of AmtB in the presence of PG, PS and PE at different lipid:protein ratios. Typical raw data are shown for the 10:1 lipid:protein ratio. The colour key applies to the whole figure. (**b**) Stabilization of AmtB by various lipids calculated using collision-induced unfolding (CIU)^8^. (**c**) Thermal denaturation of AmtB performed by CD in the presence of PG and PE, and (**d**) of AqpZ in the presence of PE and CDL. Typical raw data showing change in ellipticity at 220 nm are shown for both proteins with the different lipids. (**e**) Lipid stabilization of AqpZ measured using CIU^8^. Reported are average and s.e.m. from repeated measurements (*n=3*).

To assess the validity of our method for assessing the effect of ligand binding on membrane protein stability, it is important to compare the results from our assays with those from solution-based thermal denaturation experiments. Although these solution-based experiments cannot access the specific effects of different ligand-bound states that the MS approach can, the ensemble averages yielded by other techniques would be expected to agree with those from MS. We used both circular dichroism (CD) and differential scanning fluorimetry (DSF) to measure the effect of lipid binding on the thermal stability of the *E. coli* inner membrane proteins aquaporin Z and ammonia channel. We previously identified these proteins as having significant sensitivity to the binding of different lipids using this MS approach^8^. With CD and DSF, we did not observe the same lipid-induced protein stabilization revealed by our MS approach (Fig. 4). In these solution-phase techniques, the proteins are encased in detergent micelles, the presence of which may mask the effects of the lipids on protein stability by influencing the unfolding of the protein and thus the sensitivity of protein stability to bound lipid. We previously found that the most stabilizing lipids for these two proteins were either important for function or induced conformational changes in protein structure. On this basis, we have confidence that our method is able to identify lipids with important interactions. We conclude therefore that this MS approach is more sensitive, in particular for lipid interactions with membrane proteins, than corresponding solution-based thermal shift assays.

## Discussion

Our method provides a novel means of studying the effects of ligand binding on proteins and it is of particular utility when the system under study can bind multiple ligands, such as in the case of oligomeric proteins, where the binding events are challenging to discern using other methods. We have shown that the method can interrogate the effects on stability from ligand binding to soluble proteins and, of particular note, we demonstrate that our method can be used to characterize the effects of lipid binding on membrane proteins. To our knowledge, there are no other experiments that can provide this information in such a direct manner. The detail required to implement this method has been described and software to conduct the analysis rapidly and robustly, developed for this study, will be made widely available. Owing to the ease with which this method can be implemented, we anticipate that this methodology will find particular application to evaluating the effects of lipid binding on the stability of membrane proteins, and for drug discovery by identifying ligands with notable stabilizing effects.

## Methods

### Protein preparation

Transthyretin was a gift from Mark Pepys, UCL Division of Medicine, and streptavidin from Michael Fairhead, University of Oxford. AmtB, AqpZ and OmpF were expressed in *E. coli* and extracted from membranes using 200 mM n-octyl-β-D-glucoside. AmtB and AqpZ were purified using immobilized-metal affinity chromatography in n-dodecyl β-D-maltoside. Fusions to maltose binding protein (MBP) (for AmtB) and green fluorescent protein (AqpZ) were cleaved using tobacco etch virus protease and the cleaved proteins further purified by reverse immobilized-metal affinity chromatography^8^. OmpF was purified in n-octyl-β-D-glucoside by anion-exchange chromatography^29^. MATE was cloned from *P. furiosus* genomic DNA (ATCC 43587) into a pet15-based vector, to end up with a tobacco etch virus (TEV) protease cleavable carboxy-terminal GFP-6 × His fusion protein (equivalent to that constructed for AqpZ), expressed in C43(DE3) cells and purified with the same protocols used for AqpZ, with the exception that solubilization from the membranes used 1% (w/v) octyl glucose neopentyl glycol. Likewise, the gene for *S. aureus* MscL was cloned into the same vector backbone and purified as for AqpZ after membrane solubilization using 5% (w/v) C8E4 (ref. 30).

### Mass spectrometry

Streptavidin and transthyretin (TTR) were buffer exchanged into 300 mM ammonium acetate, pH 7.0, using a Micro Bio-Spin column (Bio-Rad) at 4 °C. Transthyretin (1.7 μM) was incubated with T4 (1.8 μM) for 4 h at room temperature, to enable equilibration of binding before MS analysis. A stock of 2 mM T4 in dimethyl sulfoxide (DMSO) was diluted to 3.6 μM in 300 mM ammonium acetate before protein addition at a ratio of 1:1; final DMSO present <0.1%, as >0.1% DMSO led to reduction in average charge and could possibly destabilize protein–protein and protein–ligand interactions. B4F was dissolved in 300 mM ammonium acetate, pH 7.0, and filtered. Streptavidin (2 μM) was incubated with B4F (2.8 μM) for 16 h at room temperature before MS analysis. The membrane proteins were prepared, after purification, for MS analysis by performing size-exclusion chromatography, to exchange into C8E4 buffer, and were subsequently exchanged into MS buffer using a centrifugal buffer exchange device^8^. All membrane proteins were sprayed in 200 mM ammonium acetate (pH 7.4) with 2 × critical micelle concentration (CMC) of the detergent C8E4.

IM mass spectra were recorded using a modified Synapt G1 instrument (Waters Corp.) as previously described^8^. The experimental concentrations of both protein and ligand used are recorded in Table 2. Measurements were made at accelerating voltage steps of 1 V for the soluble proteins and 5 V for membrane proteins, to balance experiment time and resolution.

All lipids were purchased from Avanti Lipids and were prepared as previously described^8^. The lysyl-PG used was 16:0 (1,2-dipalmitoyl-sn-glycero-3-[phospho-rac-(3-lysyl(1-glycerol))]) and was prepared in the same way.

### Circular dichroism

Thermal stability was measured by CD using a JASCO J-815 spectropolarimeter with temperature control, using 1 mM quartz cuvettes, by measuring the ellipticity at 222 nm as a function of temperature (5 °C intervals, from 20 °C to 80 °C, ramping at 5 °C min^−1^). Protein concentration was 0.76 μM for AmtB (complex), with lipid concentrations of 33 μM PE (L-α-phosphatidylethanolamine from *E. coli*) and 21 μM PG (L-α-phosphatidylglycerol from *E. coli*) in 20 mM phosphate buffer, pH 7.5, with 0.5% (w/v) C8E4. For experiments with AqpZ, the protein concentration was 1.1 μM (complex), with lipid concentrations of 210 μM PE and 11 μM CDL (cardiolipin from *E. coli*).

To calculate the *T*_m_ values, the derivatives of ellipticity as a function of temperature were calculated and fitted with a Boltzmann distribution. Repeated measurements and error given as the s.d. of the mean.

### Differential scanning fluorimetry

DSF measurements were made using an Agilent MX3005p with an ANS filter set (excitation at 330 nm and emission at 492 nm). Thermal stability was measured using CPM dye (excitation 384 nm and emission at 470 nm), which fluoresces on reaction with cysteine residues^31^. Fluorescence was measured at 1 °C intervals, as the temperature increased from 25 °C to 95 °C.

To optimize fluorescence response, three different protein concentrations (1.25, 2.5 and 5.0 μM) were assayed, each with four different lipid:protein ratios (10:1, 25:1, 50:1 and 125:1) (NB: these ratios to mole of protein complex, not monomer), for three different lipids (PG, phosphatidylserine (PS) and PE). The lipid:protein ratios used in the original MS experiments were 44:1, 28:1 and 26:1 for PE, PG and phosphatidylserine, respectively. Aside from protein and lipid, each condition contained 0.5% (w/v) C8E4, 0.2 M NaCl and 20 mM HEPES, pH 7.5. The data presented are for 1.5 μM AmtB; there were no perceptible differences in results at the other two protein concentrations. The *T*_m_ values were calculated by a similar means to that for the CD data.

### Software development

The software was developed in Python 2.7, making extensive use of the libraries scipy, numpy^32^ and matplotlib^33^. A graphical user interface developed using wxPython^34^ allows interaction with the programme. The software is a cross-platform, with the exception of data importation, which must be performed on Windows. An additional programme written in C++ is used to import the data. This uses the libraries available from Waters Corp. for accessing the proprietary binary data format of the raw files. The software is available from http://pulsar.chem.ox.ac.uk/.

### Data importation

The software can import data from Synapt IM-MS instruments (Waters Corp.). Multiple data files can be imported simultaneously into the software, and for each data file the user supplies metadata to later identify and categorize each experiment, and for the calculation of CCS values. The mass spectrum is a sum of the mass spectra in all IM scans and is twice smoothed using a moving mean algorithm, and then binned, by default every 1 *m/z*. The IM data are by default binned every 4 *m/z*, which for protein spectra is an empirically optimized bin size that gives an excellent trade-off between calculation speed and desired resolution.

### Mass spectrum fitting

To analyse the unfolding of individual ions it is first necessary to define the boundaries of each charge-state peak in the mass spectrum. These boundaries can then be used to extract the arrival time information for a particular charge state. The peaks are fitted using Gaussian functions: the overall charge-state distribution is modelled by one Gaussian function, herein called the envelope (equation (1)). *Z* is a charge state, *Z*_avg_ is the average charge state in the distribution, *w* is a width parameter and *h* a height scalar. The envelope (of a particular distribution) defines what charge states are expected to be in the spectrum and therefore what charge-state peaks should be fitted. The expected charge states within the envelope are calculated by examining 5 × *w* and selecting peaks within the boundary with a modelled intensity >0.01. The charge-state peaks themselves are also modelled by Gaussian functions (equation (2)). Here, mass is the sample mass, *Z* is the charge state of the peak, *R* is a resolution parameter and *h* is the height scalar from the envelope.




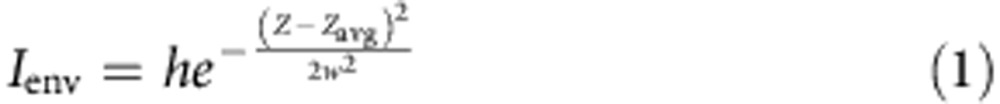







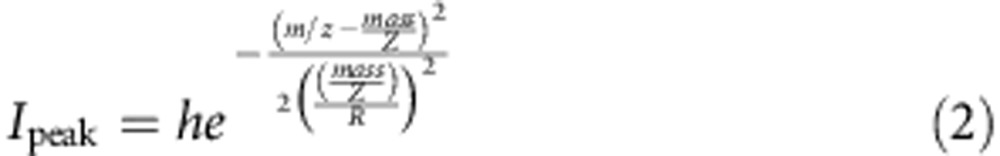




It is not necessary for the charge-state peaks to be fully resolved in the *m/z* domain to be able to achieve a useful fit to the mass spectrum; however, for analysis of the unfolding data it is required that the arrival time distribution, corresponding to a particular charge-state peak, contains data only for the ion of interest, that is, overlapping species or artefacts should not be present. Although it is possible to use more complex models to deconvolute mass spectra^16^,^35^,^36^,^37^,^38^, as the fitting is only used to determine boundaries for extracting arrival time distributions, ideal fits to the mass spectrum are not required and this simple model is entirely sufficient.

The mass spectrum fitting requires an initial guess of the mass and the charge-state value of the highest intensity ion. Although *a priori* information, for the nature of experiments performed, these values are typically already known, or easily discerned. From the initial guess, the given mass is adjusted over a range and the difference between the simulated and experimental spectra is calculated. The parameters of the model (mass, average charge state, charge-state distribution width and intensity and charge-state peak resolution) are then optimized by minimizing the difference between the simulated and experimental spectra. Alternatively, for each mass value trialed, all the parameters can be optimized before choosing that which gives the best fit. Parameters describing multiple species detected in the same mass spectrum (for example, ligand-free and ligand-bound forms of a protein) can be fitted simultaneously using the same approach. The net result is an individual theoretical spectrum for each species present in the experimental spectrum.

### Arrival time distribution extraction

To extract the arrival time distribution associated with a charge-state peak, a slice is taken of the arrival time data between two *m/z* values. The centre and hence position of the slice is known from the Gaussian function fit to the charge-state peak. For more consistent analysis, the width parameter of the modelled Gaussian function is replaced with a new resolution value, which is the same for all ions that arrival time distributions are extracted for, scaled by the *m/z* value. The width of the slice is then determined by the modified Gaussian description of the peak and an intensity threshold. The use of these two constants improves the consistency and reproducibility of the arrival time distribution extracted for each ion under investigation.

The software automatically fits a Gaussian function to the highest intensity species in each extracted arrival time distribution and multiple arrival time peaks can also be manually fitted. The centre of a modelled peak is taken as the arrival time value of a particular ion. These fits to the arrival time distribution are not used for the purposes of modelling the unfolding data; the only parameters affecting this are those used for the extraction of the arrival time distribution (*vide supra*).

### CCS calculation

It is useful to know the CCS values for species observed in the arrival time distribution and also to be able to generate unfolding plots in units of CSS as a function of accelerating voltage. The software can calculate CCS values for both travelling-wave and drift-tube IM data.

For travelling-wave IM measurements, a calibration curve is necessary to calculate CCS values. The calibration curve is created by measuring the arrival times of standards with known CCS values, under the same instrument conditions used for the sample under investigation^39^. To facilitate calibration curve creation, the software has a module for automated processing of calibration spectra. Using the same approach as outlined above, the mass spectra for each calibration sample is fitted and the arrival time distribution extracted for charge states with known CCS values. The peaks in the arrival time distribution are automatically fitted, yielding peak centres corresponding to the arrival times for each ion. Multiple calibration samples, measured under common instrument conditions, are then combined to form a calibration curve. The resulting calibration file can then be selected and used for the computation of CCS values of the sample.

For measurements made using drift-tube instruments, CCS values are directly calculated from arrival time measurements using the Mason–Schamp equation^39^,^40^. This requires calculation of the drift time, which is calculated from the arrival time by subtracting the value of *T*_0_, which is instrument, settings and ion specific. The value of *T*_0_ must be separately measured and calculated^41^. The software provides a module for calculating the value of *T*_0_. An advantage of this approach to calculate CCS values is that the number of necessary measurements is greatly reduced, as instead of requiring measurements at multiple drift voltages for each accelerating voltage only one is necessary.

### Quantification of collision-induced unfolding

Collision-induced unfolding experiments are carried out by systematically increasing the accelerating voltage to progressively unfold the protein. The unfolding plot is constructed by stacking the intensity-normalized arrival time distributions at each accelerating voltage for a particular charge state.

The software models the gas-phase unfolding by means of a simple protein unfolding model (equations (3, 4, 5))^17^, which describes the conformational stability of the species as a function of accelerating voltage and can be extended to any number of species.




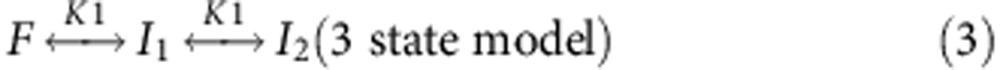







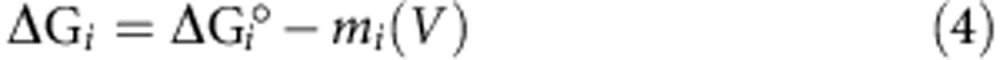







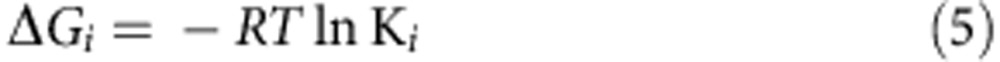




Initially, the positions and widths of each species (for example, folded, intermediate one and intermediate two) are defined by CCS ranges. The software provides the ability to load saved-species parameters to allow the initial definitions to be re-used for subsequent analyses and between different experiments.

The fitting of the unfolding model to the data proceeds as thus: the measured arrival time intensities for each species are summed and normalized at each accelerating voltage step. Next, the unfolding model is fitted to these data using a choice of minimization algorithm, yielding initial thermodynamic parameters. These initial values are then used as a seed for a more complex fitting, where a theoretical unfolding plot is back-calculated and the difference to the experimental unfolding plot minimized.

The theoretical unfolding plot is generated by defining a Gaussian function for each species at every accelerating voltage, where the width and origin of each Gaussian begin as those from the definitions derived from the original species range. From the initial unfolding model seed, the fraction (*F*) of each species present at each accelerating voltage value can be calculated (equation (6, 7, 8, 9)) and is then used to scale the height of the Gaussian function for that species. Finally, because the intensities in the experimental unfolding plot are normalized at each accelerating voltage, the theoretical unfolding plot is normalized in the same way. The difference between the resulting theoretical unfolding plot and the experimental unfolding plot is then minimized.




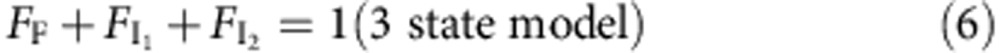







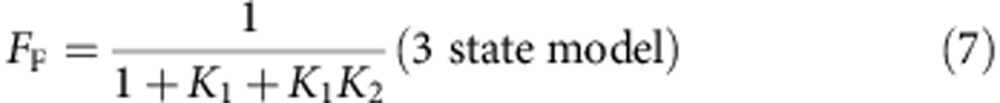







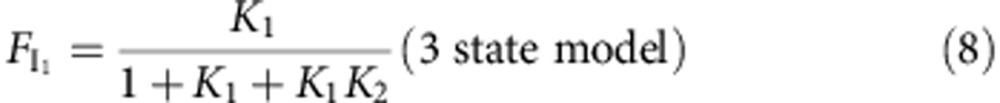







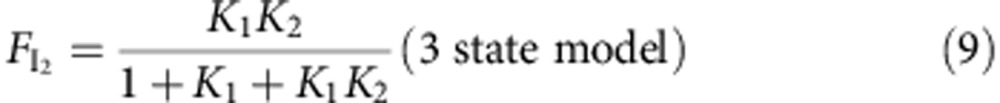




There are three approaches available for fitting the unfolding model to the experimental data. First, a ‘basic’ fit can be used where only the thermodynamic parameters of the unfolding model are fitted using fixed values for the CCS position and width for each species as provided by the user. A ‘semi’ approach fits both the thermodynamic parameters and the position, but using the fixed width of each species defined by the user. Finally, the ‘full’ approach fits all parameters and typically gives the best fits to the experimental data, which we strongly recommend for calculating ligand-induced protein stabilization.

To make the calculations more efficient, the unfolding plot data are trimmed to within 10% of the minimum and maximum CCS values defined by the native-like and last unfolding species, respectively. Trimming the data typically results in on average around 900 data points.

As the fitting surface can be rugged, a sophisticated minimization procedure to maximize the chance of finding a global minimum is required. First, the model is minimized using the Powell’s conjugate direction method available in scipy. This provides the boundaries for bio‐inspired algorithms; all parameters are set to boundaries of ±25% with the exception of the CCS species peak centres, which are set to ±5%. Models are minimized using the modified differential algorithm (de_1220) available in PyGMO using the following parameters: population of 20, 12 evolutions, 250 generations and 8 islands. Other algorithms, such as particle swarm and bee colony typically produce similar or lower *R*^2^-values and are computationally more expensive.

Successful fitting of the experimental data lead to thermodynamic parameters for each species, which enables the quantification of unfolding transitions and ligand-induced protein stabilization. The transition midpoint is where 50% of a specific transition state *i* is depleted. This metric removes correlations between 
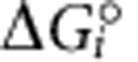
 and *m* inherent in the fitting procedure. To average out any systematic and/or variability in droplet formation, stabilization should be calculated as given in equation (10), which is the sum of the differences in midpoint values between ligand-bound and -unbound forms, which we normalize for the number of transitions (*i*_max_), and can be converted to laboratory frame energy by multiplying by the charge state.




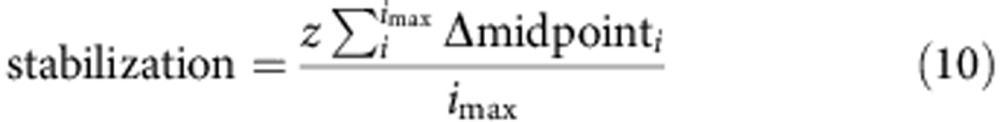




### Bootstrapping analysis

The ability to perform bootstrapping analysis accompanies the main software in the form of external scripts that facilitate the analysis to be performed in clustered computing environments. The sampling is by replacement, where accelerating voltage slices of the unfolding plots are randomly chosen. As stabilization is calculated from the modelling of two different unfolding plots (ligand free and ligand bound), the bootstrapping calculates a sampling schedule that will be the same for each stabilization calculation. Model parameters from fits to the original data are used as the initial parameters to the fitting of each sample. Typically, the sampling is repeated 100 times, which is sufficient for the convergence of stabilization values and model parameters.

## Additional information

**How to cite this article:** Allison, T. M. *et al*. Quantifying the stabilizing effects of protein–ligand interactions in the gas phase. *Nat. Commun.* 6:8551 doi: 10.1038/ncomms9551 (2015).

## Supplementary Material

Supplementary Movie 1 IM-MS spectra processing and application of the methodology to quantify gas-phase ligandinduced protein stabilization using the software PULSAR. The main features of PULSAR are shown with some example collision-induced unfolding data. Data importation, mass assignment, unfolding plot generation, and ligand-induced gas-phase stabilization calculation are highlighted.

## Figures and Tables

**Table 1:** Bootstrapping analysis of transthyretin-thyroxine stabilization.

**Repeat**	**1 × T4**	**1 × T4 bootstrap**	**2 × T4**	**2 × T4 bootstrap**
1	48.8	54.9±11.8	64.2	63.5±11.5
2	60.5	48.4±6.1	76.2	79.1±8.2
3	59.7	56.5±6.5	75.1	77.5±9.8
Average	56.3±5.3	53.3	71.8±5.4	73.4

Bootstrapping was performed using sampling with replacement (*n*=100) of collision-voltage slices of unfolding plots. The model parameters for each sample were initiated to those calculated for the original data. Errors shown are s.d.

**Table 2:** Concentration of proteins and ligands used for collision-induced unfolding experiments.

**Protein**	**[Protein] (μM)**	**Ligand**	**[Ligand] (μM)**
*Ec*AmtB	See^8^		
*Ec*AqpZ	See^8^		
*Ec*OmpF	2.9	Rough LPS	NA
*Pf*MATE	3.8	CDL	7.5
	3.8	PG	14
*Sa*MscL	7.3	PG	14
	4.6	CDL	7.0
	3.7	Lysyl-PG	10
Streptavidin	2.0	B4F	2.8
Transthyretin	1.7	T4	1.8

B4F, biotin-4-fluorescein; CDL, cardiolipin; *Ec*OmpF, β-barrel outer-membrane protein OmpF from *Escherichia coli*; LPS, lipopolysaccharide; *Pf*MATE, multi-antimicrobial extrusion protein from *P. furiosus*; PG, phosphatidylglycerol; NA, not applicable; *Sa*McsL, mechanosensitive channel of large conductance from *Staphylococcus aureus*; T4, L-thyroxine.
